# Quinones as Neuroprotective Agents

**DOI:** 10.3390/antiox12071464

**Published:** 2023-07-20

**Authors:** Ángel Cores, Noelia Carmona-Zafra, José Clerigué, Mercedes Villacampa, J. Carlos Menéndez

**Affiliations:** Unidad de Química Orgánica y Farmacéutica, Departamento de Química en Ciencias Farmacéuticas, Facultad de Farmacia, Universidad Complutense, Plaza de Ramón y Cajal sn, 28040 Madrid, Spain; acores@ucm.es (Á.C.); noecarmo@ucm.es (N.C.-Z.); joscleri@ucm.es (J.C.); mvsanz@ucm.es (M.V.)

**Keywords:** oxidative stress, Keap1/Nrf2 pathway, covalent drugs, coenzyme Q_10_, idebenone, vatiquinone, embelin, pyrroloquinolinequinone, memoquin, multitarget drugs

## Abstract

Quinones can in principle be viewed as a double-edged sword in the treatment of neurodegenerative diseases, since they are often cytoprotective but can also be cytotoxic due to covalent and redox modification of biomolecules. Nevertheless, low doses of moderately electrophilic quinones are generally cytoprotective, mainly due to their ability to activate the Keap1/Nrf2 pathway and thus induce the expression of detoxifying enzymes. Some natural quinones have relevant roles in important physiological processes. One of them is coenzyme Q_10_, which takes part in the oxidative phosphorylation processes involved in cell energy production, as a proton and electron carrier in the mitochondrial respiratory chain, and shows neuroprotective effects relevant to Alzheimer’s and Parkinson’s diseases. Additional neuroprotective quinones that can be regarded as coenzyme Q_10_ analogues are idobenone, mitoquinone and plastoquinone. Other endogenous quinones with neuroprotective activities include tocopherol-derived quinones, most notably vatiquinone, and vitamin K. A final group of non-endogenous quinones with neuroprotective activity is discussed, comprising embelin, APX-3330, cannabinoid-derived quinones, asterriquinones and other indolylquinones, pyrroloquinolinequinone and its analogues, geldanamycin and its analogues, rifampicin quinone, memoquin and a number of hybrid structures combining quinones with amino acids, cholinesterase inhibitors and non-steroidal anti-inflammatory drugs.

## 1. Introduction

### 1.1. General Features of Neurodegenerative Diseases

Neurodegenerative diseases have become a major challenge for healthcare systems, as they show a strong correlation with age and life expectancy is rising worldwide [1]. Their multifactorial origin hampers the discovery of new effective treatments, and current available therapies only procure symptomatic relief to some extent, without modifying the clinical course [2].

Although some hypotheses have been traditionally proposed to explain the etiopathology of neurodegenerative diseases—e.g., cholinergic hypothesis for Alzheimer´s disease or dopaminergic dysfunction for Parkinson´s disease, recent research in this field attributes these pathologies to several alterations, generally connected to aging and genetic features. Thus, several phenomena such as oxidative stress, proteinopathies, neuroinflammation and excitotoxicity may be the real events causing neurodegeneration and cognitive/motor decline [3,4]. 

**Figure 1:**
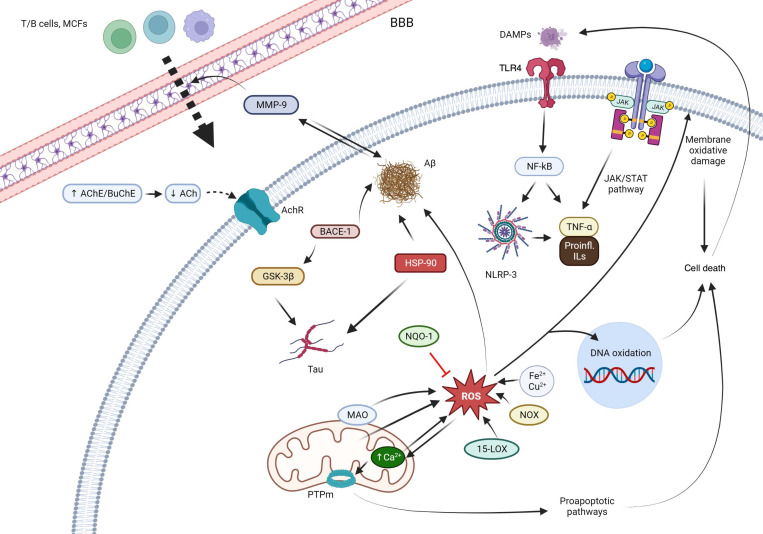
Main mechanisms of action of the quinones described in this review. Abbreviations: T/B cells: the two main types of lymphocytes. BBB: blood brain barrier. AChE: acetylcholinesterase. BChE: butyrylcholinesterase. AChR: acetylcholine receptor. MMP-9: matrix metalloproteinase 9. Aβ: beta-amyloid protein. BACE-1: beta-secretase 1. GSK-3β: glycogen synthase kinase-3 beta. HSP90: Heat-shock protein 90. NQO1: NAD(P)H:quinone oxidoreductase 1. ROS: reactive oxygen species. MAO: monoamino oxidase. PTPm: mitochondrial permeability transition pore. 15-LOX: 15-lipooxygenase. NOX: nitric oxide. DAMPs: damage-associated molecular patterns. TLR4: Toll-like receptor 4. NF-κB: Nuclear factor kappa-light-chain-enhancer of activated B cells. NRLP3: NLR family pyrin domain containing 3. JAK: Janus kinase. STAT: signal transducer and activator of transcription. TNF-α: tumor necrosis factor alpha. IL: interleukins.

Oxidative stress seems to have a key role in neurodegeneration, both by causing direct oxidative damage to lipids, proteins and DNA and by providing feedback to other pathological mechanisms. Oxidative damage of proteins causes them to misfold and aggregate into toxic oligomers or deposits that consequently activate an inflammatory response that aggravates the oxidative status. Mitochondrial DNA also suffers modifications by oxygen and nitrogen reactive species, giving rise to dysfunctions in key proteins involved in cellular metabolism. Thus, mitochondrial activity is impaired, resulting in increased ROS production levels and activation of proapoptotic pathways. Lipid peroxidation by reactive species disrupts the structure and permeability of cellular membranes, leading to inflammation, alterations of calcium homeostasis and neuronal death. These mechanisms are only some representative examples of how oxidative stress plays a central role in the pathological network that underlies neurodegeneration [5,6,7]. Figure 1 summarizes the pathological mechanisms that are interfered by the quinones described in the present review.

Treatments currently employed against neurodegenerative diseases mainly target events such as neurotransmitters deficit, whose alteration is considered nowadays as a consequence of aforementioned phenomena, rather than as the primordial cause of neurodegeneration. Thus, all the efforts are being directed to the discovery of disease-modifying therapies, that may impact on the real causes of these diseases [8]. 

Quinones are a family of natural and synthetic compounds characterized by having a fully conjugated cyclic unsaturated dione structure. Quinones have been extensively studied for their biological activities, especially as chemotherapeutic agents [9,10,11]. Moreover, some endogenous quinones, most notably coenzyme Q_10_, show interesting neuroprotective properties, which has prompted the inclusion of quinone structural fragments as a strategy in drug discovery in this area. Quinones, as it will be comprehensively discussed in this review, exhibit powerful redox and antioxidant properties, which can be addressed to interfere in the oxidative status of stressed or senescent cells, aiming to interrupt the vicious circle of oxidative stress, neuroinflammation and protein misfolding.

In this context, we review here the use of quinones as neuroprotective agents, potentially useful for the treatment of neurodegenerative diseases. A prior review of neuroprotection by quinones, more narrowly focused and dealing only with the use of naphthoquinones and anthraquinones against Alzheimer’s disease, has been recently published [12].

### 1.2. General Features of Quinone Chemistry Relevant to Neuroprotection

**Figure 2:**
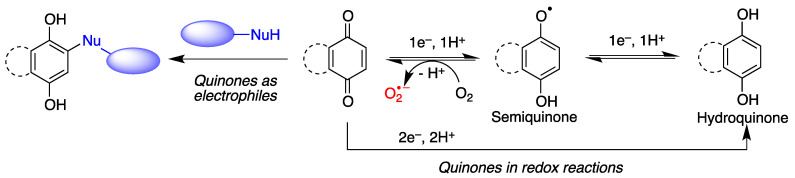
General features of quinone chemistry of relevance to neuroprotection. Quinones may act as electrophiles, thereby alkylating proteins or nucleic acids. Additionally, they can accept one electron to give semiquinones, which can in turn be reduced to hydroquinones by one-electron transfer and protonation. Semiquinone formation can be reverted by molecular oxygen, leading to the generation of superoxide anion-radicals.

Quinones are often cytoprotective but they can also create a variety of toxic effects, including acute cytotoxicity and immunotoxicity and can therefore be viewed as a double-edged sword in cytoprotection. Although other targets may be involved, these apparently contradictory responses to quinones are generally driven by their chemical properties, summarized in Figure 2.

(a) Quinones are redox active molecules that can form semiquinone radicals by a reversible one-electron transfer process that in the presence of oxygen generates superoxide, a reactive oxygen species in the reverse reaction (Figure 2). Thus, quinones can cause oxidative stress leading to the oxidation of lipids, proteins and DNA.
(b) Quinones are Michael acceptors and thus can cause cellular damage by alkylating cellular proteins, especially at cysteine residues, or DNA (Figure 2).
(c) Due to their planar structure and their ability to form hydrogen bonds, many quinones are able to bind to amyloid proteins, preventing their aggregation and allowing the visualization of diffuse and dense-core amyloid plaques [13]. Molecular dynamics simulations have shown that anthraquinone, used as a model simple quinone, interferes with the early phase of aggregation by intercalation into the β-sheet of the hydrophobic H14QKLVFF20 sequence that promotes Aβ self-assembly. The amyloid-quinone complex is formed via polar interactions with the peptide backbone, which destabilize interstrand hydrogen bonds [14].

### 1.3. Quinones as Redox Modifiers of Biomolecules

Quinones are pro-oxidant due to their ability to take part in one-electron transfer processes, leading to semiquinones that can then revert to the original quinone by oxidizing a molecule of oxygen to superoxide. This reaction is the basis of the anticancer properties of quinone natural products such as the anthracyclines. 

**Figure 3:**
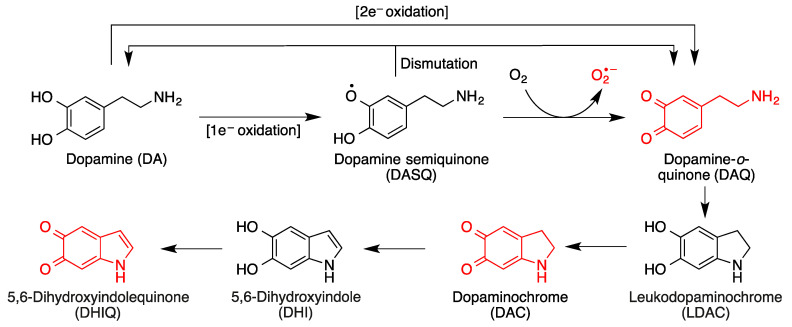
Toxic species derived from the oxidative metabolism of dopamine. Formation of dopamine-*o*-quinone (DAQ) from the one-electron or two-electron oxidation of dopamine and its evolution by intramolecular Michael additions and additional oxidation reactions.

On the other hand, by action of NQO1, an inducible enzyme that catalyzes their two-electron reduction, quinones are transformed into the corresponding antioxidant hydroquinone forms. It is relevant to note that NQO1 levels are increased in patients of diseases that involve high levels of oxidative stress, such as Alzheimer’s disease. In certain situations (e.g., oxidative stress), hydroquinones can be transformed back into quinones, which can lead to cytotoxic responses. One relevant example in the area of neurodegeneration is dopamine, which is a catechol and therefore a hydroquinone (Figure 3). It is the most abundant catecholamine in the brain, and serves as the biosynthetic precursor to other neurotransmitters.

In situations of oxidative stress such as those prevalent in most neurodegenerative diseases, cytosolic dopamine can generate a variety of neurotoxic species in the brain, including three quinones [15,16] (Figure 3). Dopamine oxidation products act as mitochondrial endotoxins, providing a potential mechanism for preferential neurodegeneration of dopamine-containing neurons in Parkinson’s disease [17], and also have additional toxicities relevant to the onset and progress of Parkinson’s disease, such as impaired protein degradation, mitochondrial dysfunction and α-synuclein aggregation [18].

**Figure 4:**
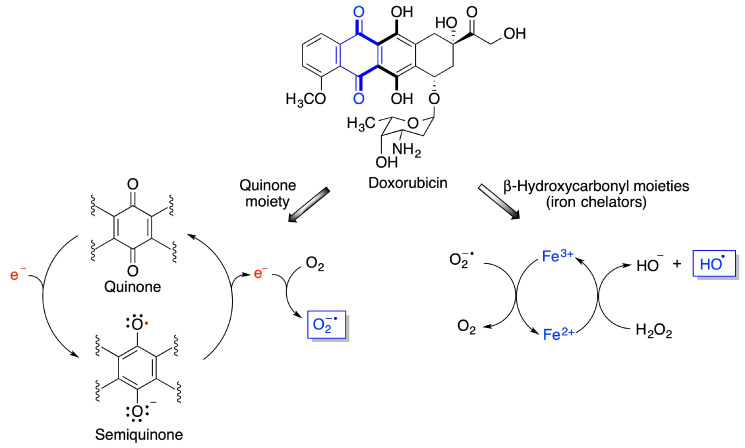
Mechanisms explaining the generation of reactive oxygen species from doxorubicin.

Other quinones, including environmental toxins such as polychlorinated biphenyl (PCB) quinones have been shown to cause severe cellular oxidative stress [19,20]. One particularly interesting case is that of doxorubicin, a member of the anthracycline class of anticancer drugs [21]. Doxorubicin undergoes redox cycling to a semiquinone species that reverts back to the quinone form by one-electron transfer to a molecule of oxygen that becomes superoxide anion, one of the reactive oxygen species (ROS). On the other hand, doxorubicin is also a metal chelator, due to the presence of two γ-hydroxycarbonyl moieties in its structure. Due to ionic interactions with the DNA phosphate groups, the anthracycline-Fe^3+^ chelate tightly binds to DNA. When Fe^3+^ reacts with the superoxide anion generated by the quinone moiety, it becomes Fe^2+^ that forms hydroxyl radicals via its Fenton reaction with hydrogen peroxide (Figure 4). Doxorubicin causes substantial heart damage due to oxidative stress [22,23] and it is also connected to its neurotoxicity, as will be discussed below.

**Figure 5:**
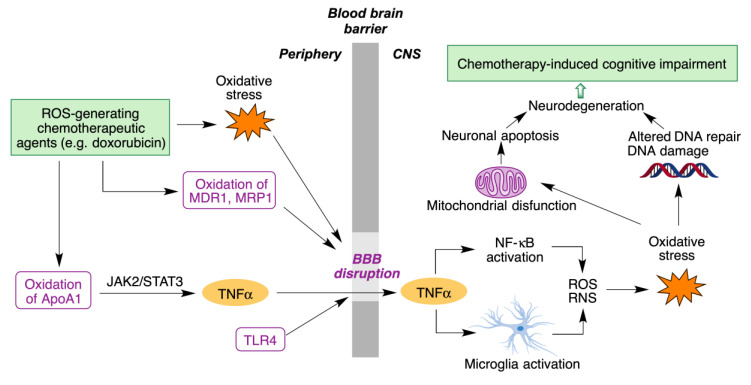
Main molecular mechanisms involved in chemotherapy-induced cognitive impairment.

Although doxorubicin cannot cross the blood-brain barrier (BBB), it nevertheless leads to chemotherapy-induced cognitive impairment (CICI), described by patients as “chemobrain” or “chemofog”. CICI is associated with decreased learning abilities, memory and attention capacity that greatly impair the quality of life of many cancer survivors. The complex molecular mechanisms of CICI are initiated by oxidative stress, resulting in protein oxidation and lipid peroxidation, and hence increased levels of the tumor necrosis α factor (TNF-α). On the other hand, activation of the toll-like receptor 4 (TLR4) also contributes to BBB disruption and production of TNF-α and other cytokines. TNF-α is able to cross BBB due to the oxidative stress-mediated disruption of the latter. Once inside the brain, TNF-α triggers local immune response by microglia and NF-κB activation, accompanied by the production of reactive oxygen and nitrogen species (ROS/RN). The subsequent oxidative stress affects DNA repair systems, leading to neurodegeneration. It also affects mitochondria function, with opening of the mitochondria permeability transition pore (mPTP) and release of cytochrome C. This initiates the caspase signaling process, eventually leading to neural apoptosis (Figure 5) [24].

### 1.4. Quinones as Covalent Modifiers of Biomolecules

**Figure 6:**
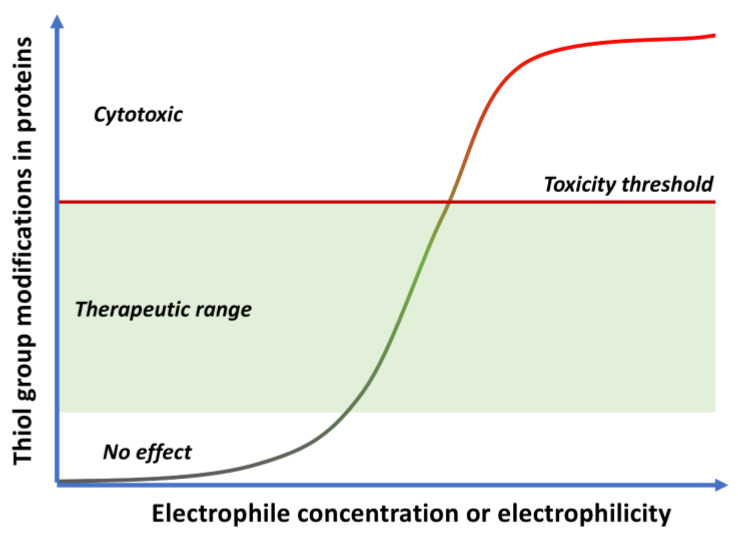
Cytoxic vs. cytoprotective responses to quinones, depending on their concentration and electrophilicity.

Electrophilic compounds, such as quinones, have traditionally had a reputation for toxicity, although covalent drugs are currently undergoing a renaissance and indeed about 30% of the drugs marketed in the last few years belong to this class [25]. As other covalent modulators, quinones can show opposing effects leading to cytoxicity or cytoprotection [26,27] depending on the dose, exposure time and intrinsic electrophilicity of the quinone, as shown in Figure 6. 

**Figure 7:**
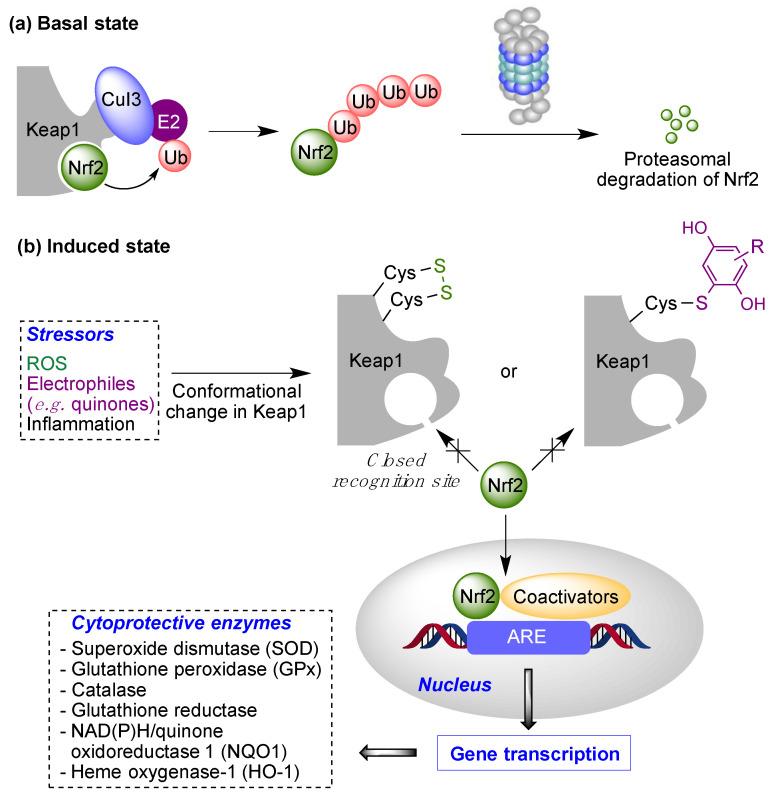
The Keap1/Nrf2 cytoprotective pathway. Basal state: Negative regulation of Nrf2 under normal conditions (“basal state”) and its activation under pathological conditions (“induced state” in the presence of reactive oxygen species (ROS) or covalent modifiers.

Low doses of moderately electrophilic quinones, which in structural terms are those that are tetrasubstituted or trisubstituted with electron-releasing substituents, are generally cytoprotective. The level of stimulation that they bring about by covalent alteration of biomolecules and by the induction of mitochondrial or cytosolic ROS formation (see Section 1.2) triggers an adaptive stress response. This response arises mainly from the ability of both electrophiles and oxidants to activate the Keap1/Nrf2 pathway, the key regulator of the phase II antioxidant response, resulting in the induction of the expression of several detoxifying enzymes [28]. Under physiological, non-stress conditions, Nrf2 is located in the cytosol associated to Keap1 (Kelch-like ECH-associated protein 1), the main negative regulator of Nrf2. Keap1 is able to generate a complex with Cul3, an E3-ubiquitin ligase, inducing Nrf2 ubiquitination and thus its proteasomal degradation [29]. In oxidative stress situations, several cysteine residues (Cys151, Cys273, Cys288 and Cys297) present in the “sensor” region at the C-terminal domain of Keap1 are oxidized to disulfides, which causes a conformational change that releases Nrf2 from Keap1. These cysteine residues can also be activated by reaction with electrophiles, including quinones. After its liberation, Nrf2 translocates into the nucleus, where, after forming heterodimers with several coactivators such as the small musculoaponeurotic fibrosarcoma protein (sMaf), it promotes the transcription of the ARE (antioxidant response element) region in DNA. This region encodes several enzymes able to scavenge ROS and neutralize electrophiles, including superoxide dismutase (SOD), glutathione peroxidase (GPx), catalase, glutathione reductase, NAD(P)H/quinone oxidoreductase 1 (NQO1) and heme oxygenase-1 (HO-1) [30] (Figure 7). Other protective pathways are known to be activated by quinones, including the heat-shock transcription factor-1 (HSF-1) that induces heat-shock proteins that protect from endoplasmic reticulum (ER)-related stress [31]. 

On the other hand, the administration of more reactive quinones, or high doses of moderately reactive ones, can lead to toxic effects associated to the depletion of glutathione (GSH), a defense mechanism against electrophiles, and the formation of covalent adducts of the quinone to proteins or DNA. In the latter case, long-term exposure may lead to DNA alterations that are not repaired efficiently and can therefore promote mutations and carcinogenesis. Finally, very reactive quinones are likely to react with water before reaching any biomolecule, having therefore little biological effect, or bind only to the oxidative enzymes that generate them (e.g., cytochromes).

One aspect of electrophilic drugs, including quinones, that needs to be addressed at the discovery stage is the possibility that, by reacting indiscriminately against all kinds of proteins, they act as pan-assay interference compounds (PAINS), giving false positives in the drug discovery process [32]. It is nevertheless relevant to note the recent calls for caution against the blind use of PAINS filters [33]. 

## 2. Endogenous Quinones and Their Analogues

Several quinones are involved in physiological processes, normally taking part in one-electron transfer processes, as will be discussed below. Synthetic analogues of these natural molecules will also be included in the discussion.

### 2.1. Coenzyme Q_10_ (CoQ, Ubiquinone)

An example of a 1,4-benzoquinone with a remarkable relevance to physiological processes is coenzyme Q_10_ (CoQ), or ubiquinone 10, which takes part in oxidative phosphorylation processes involved in cell energy production, acting as a proton and electron carrier in the mitochondrial respiratory chain [34]. It also has some relevant extramitochondrial roles, acting as a recycler of the oxidized form of tocopherols thanks to its antioxidant properties [35]. It is also an inflammasome regulator, therefore having anti-inflammatory effects [36], and a modulator of autophagy [37].

**Figure 8:**
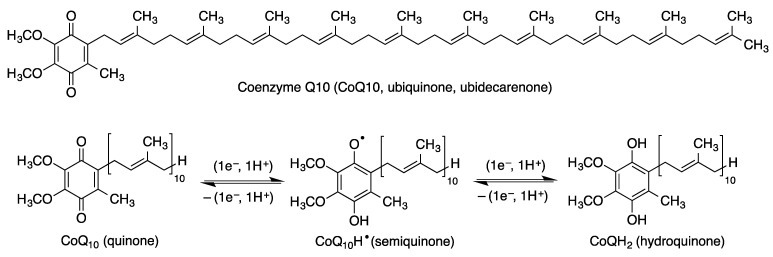
Structure of coenzyme Q_10_ and generation of its three oxidation states by two successive one-electron reductions.

Coenzyme Q_10_ also contributes to the protection of phospholipids in cell membranes, including mitochondrial membranes, and lipoproteins, against the phenomenon of lipid peroxidation. The actual antioxidant species is the coenzyme Q_10_ hydroquinone (CoQ_10_H_2_), which is generated by enzymes present in the cell membrane such as NAD(P)H oxidoreductases via a process having a semiquinone (CoQ_10_H^·^) as an intermediate. Thus, coenzyme Q_10_ can exist in the three oxidation states shown in Figure 8, namely the quinone, semiquinone and hydroquinone species, that are transformed into each other by one-electron transfer reactions.

**Figure 9:**
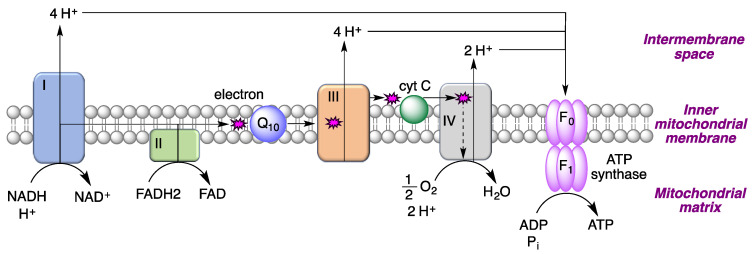
The role of coenzyme Q_10_ in electron and proton transfer processes in the mitochondrial respiratory chain.

The ability of coenzyme Q_10_ to accept and then release single electrons explains its participation in the mitochondrial electron transport chain. Thus, coenzyme Q_10_ present in the inner mitochondrial membrane accepts electrons from reducing equivalents arising from metabolism and transfers them to electron acceptors such as mitochondrial complex III. It also contributes to the transfer of protons from the mitochondrial matrix to the intermembrane space by proton pumps (complexes I, II and III), leading to a proton gradient across the inner mitochondrial membrane and hence to the generation of the mitochondrial membrane potential (ΔΨ_m_). This gradient serves as a form of storage of energy, which is released when the protons flow back into the mitochondrial matrix and used to form ATP (Figure 9).

**Figure 10:**
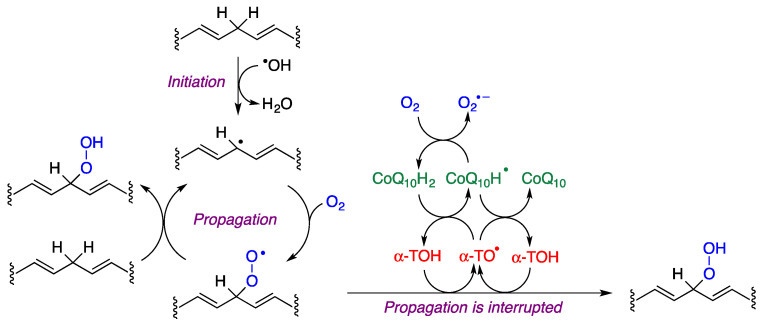
The lipid peroxidation process and mechanisms that protect against it involving the interplay of α-tocopherol and coenzyme Q_10_.

The peroxidation of unsaturated lipids is initiated by the formation of lipid radicals, which are transformed into lipid peroxyl radicals (ROO·) by reaction with oxygen. These radicals diffuse easily in the membrane and can react with intact molecules of unsaturated lipids, closing a catalytic cycle. α-Tocopherol (α-TOH) is the main antioxidant present in biological membranes and reacts with peroxyl radicals about 1000 times faster than peroxyl radicals, halting the propagation of radicals in the membrane and in lipoproteins. In this reaction, the hydroxyl group of tocopherol becomes oxidized, being transformed into a radical (α-TO·), and therefore a mechanism that regenerates the reduced form of the antioxidant (α-TOH) is necessary. This is achieved by the reduced form of coenzyme Q10 (CoQ_10_H_2_), which in the process is transformed into its semiquinone form (CoQ_10_H·), which is also able to reduce a molecule of α-TO· to the active form (α-TOH). Alternatively, CoQ_10_H· can react with oxygen to produce superoxide, which is an oxidizing agent although much less active than ROO· (Figure 10).

Beyond free radical scavenging, the neuroprotective effects of coenzyme Q_10_ have been ascribed, at least in part, to Nrf2 induction due to the presence of electrophilic Michael acceptors in the quinone moiety [38,39]. Additional experiments have confirmed that it also has anti-inflammatory effects, decreasing the protein and mRNA levels of pro-inflammatory cytokines, and also that it shows anti-apoptotic properties [40].

**Figure 11:**
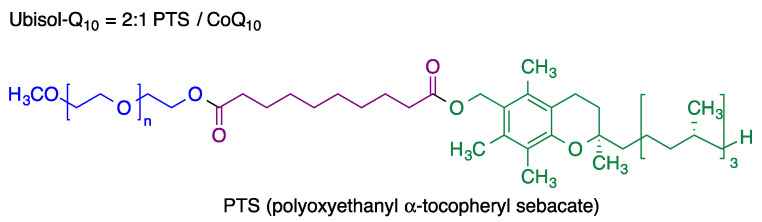
Structure of ubisol-Q_10_, a nanomicellar formulation combining coenzyme Q_10_ with polyoxyethanyl α-tocopheryl sebacate (PTS).

Recent research on coenzyme Q10 shows its potential neuroprotective effect in Alzheimer’s disease, since it is able to prevent induced neural stem cell death induced by the β-amyloid (Aβ) protein [41] and improves memory in aged rodents [42]. Furthermore, low Q_10_ levels have been correlated with a greater risk of dementia in a study performed on the Japanese general population [43]. Coenzyme Q_10_ has shown neuroprotective activity in Parkinson’s disease since it protects against mitochondrial damage and, used as prophylactic treatment, it can slow the disease progression [44]. Furthermore, it has been confirmed that CoQ10 prevents damage to dopaminergic neurons induced by 6-OHDA, reduces the levels of matrix metalloproteinase (MMPs) preventing further damage to the brain blood barrier (BBB), increases the levels of tyrosine hydroxylase and improves motor function [45]. It has a low oral bioavailability, which is a serious limitation for its therapeutic use. This obstacle may be circumvented with continuous, intrastriatal administration of a low dose of CoQ_10_ [46], although this approach does not seem very practical. As an alternative, a nanomicellar, water-dispersible formulation of coenzyme Q_10_ known as Ubisol-Q_10_ has been designed [47]. It consists of a 1:2 mixture of coenzyme Q_10_ with polyoxyethanyl α-tocopheryl sebacate (PTS), a self-emulsifying amphiphilic derivative of vitamin E (Figure 11). This formulation showed potent neuroprotective effects and was effective when delivered either prior or after neurotoxin exposure [48].

### 2.2. Coenzyme Q10 Analogues

Many coenzyme Q_10_ analogues have been synthesized in an effort to improve its bioavailability and drug-like properties [49]. These compounds have been designed either by modifying the hydrophobic tail of coenzyme Q_10_ or by altering the substituents at the quinone moiety.

#### 2.2.1. Idebenone

**Figure 12:**
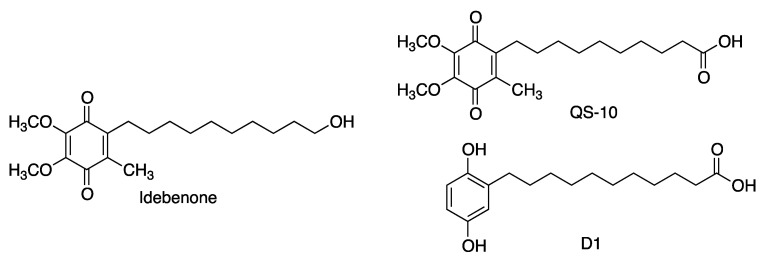
Structures of idebenone, its active metabolite, QS-10 and the related hydroquinone D1.

Idebenone is a synthetic compound having the same quinone core as coenzyme Q10 but a shorter, less lipophilic tail containing 10 carbon atoms (as opposed to the 50 carbons in Q10) and a terminal hydroxy group. Idobenone has a much better oral absorption than Q10, but also a very fast metabolism, being undetectable in plasma 1 h after administration. For this reason, its biological properties have been ascribed to its metabolites, such as the carboxylic acid QS-10 (Figure 12) [50,51]. It is relevant to note that compound D1, a precursor of a quinone structurally related to QS-10, exerts neuroprotection by a dual mechanism involving activation of the Keap1/Nrf2 pathway and also of the heat-shock transcription factor-1 (HSF-1), thereby inducing the levels of heat-shock proteins [31].

Idebenone is a substrate for the mitochondrial complexes II and III, but, unlike CoQ_10_, it is not a good substrate for complex I and in fact it inhibits its activity via occupation of the CoQ_10_ binding site. In spite of complex I blockade, the respiratory chain is not inhibited because idobenone can elicit electron transport to complex III by a mechanism involving the reduction of idobenone to its hydroquinone by NADH-quinone oxidoreductase 1 (NQO1), followed by entrance of this hydroquinone into mitochondria, where it is directly oxidized by complex III. Besides its effect on the mitochondrial respiratory chain, idebenone is a potent intracellular antioxidant. The antioxidant species is the reduced hydroquinone form, which is generated by the action of cytoplasmic NQO1, not requiring, as is the case for other quinones, the activity of mitochondrial respiratory complexes for its activation. Moreover, brain NQO1, expressed primarily by glia, enables idebenone to shuttle reducing equivalents from cytoplasmic NAD(P)H to mitochondrial complex III, bypassing any upstream damage to the electron transport chain [52].

Due to its ability to bypass complex I deficiency by directly transferring electrons to complex III, idebenone can be expected to be a better treatment than CoQ_10_ for complex I-related diseases. One of them is Leber’s hereditary optic neuropathy (LHON), which is associated to the death of retinal ganglion cells due to impairment of complex I. Idebenone has been shown to be the best antioxidant to treat LHON patients and a clinical trial showed its ability to promote the recovery of visual acuity in most patients, even after discontinuation of the treatment [53,54]. Other neurodegenerative diseases that are associated to complex I deficiency and have shown to be susceptible to treatment with idebenone include the Leigh syndrome, mitochondrial encephalomyopathy, lactic acidosis and stroke-like episodes (MELAS), Duchene muscular dystrophy and glaucoma. It is also under clinical assay for Friedreich ataxia (FRDA) [55], multiple sclerosis [56] and Parkinson’s disease [57].

#### 2.2.2. Decylubiquinone and Mitoquinone

**Figure 13:**
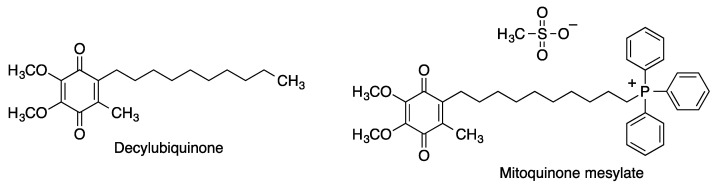
Structures of decylubiquinone and its mitochondria-targeted phosphonium analogue mitoquinone mesylate.

Decylubiquinone (DUb) is a CoQ_10_ analogue at which the isoprenoid side chain of the natural quinone has been replaced by a saturated decyl hydrocarbon chain that favors its retention by the mitochondrial membranes. Mitoquinone (mitoQ) is a mitochondria-targeted CoQ10 analogue designed by incorporating to DUb a triphenylphosphonium moiety, which due to its inherent positive charge accumulates selectively within mitochondria, driven by the mitochondrial membrane potential (Figure 13). Once in the mitochondrial matrix, the quinone moiety of MitoQ is reduced by complex II yielding the corresponding hydroquinone. This is a potent antioxidant that protects the components of the mitochondrial electron transport chain from lipid peroxidation, itself becoming oxidized in the process but being repeatedly recycled to the active reduced form by the respiratory chain.

Mitoquinone has been widely studied against neurodegenerative diseases. It has shown good activity in several in vitro models of Parkinson’s and Alzheimer’s diseases and it prevented cognitive decline in a mouse model of AD. There are also many clinical trials on humans related to the treatment of Parkinson’s disease [58] and multiple sclerosis [59].

#### 2.2.3. Plastoquinone and Its Analogues

**Figure 14:**
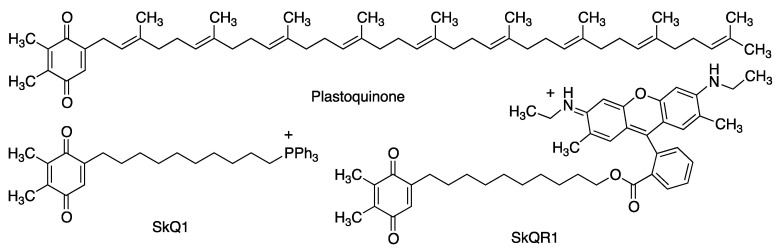
Structure of plastoquinone and some of its synthetic analogues, namely the phosphonium derivative SkQ,1 designed for mitochondrial penetration and retention, and the fluorescent plastoquinone-rhodamine hybrid SkQR1.

Plastoquinone (PQ-9) is a natural quinone, structurally close to coenzyme Q_10_, that acts as an essential electron carrier in photosynthesis and also serves for ROS removal in the chloroplast, which is subject to high levels of oxidative stress [60]. The phosphonium derivative SkQ1 was designed in order to achieve a plastoquinone analogue able to penetrate and be retained in the mitochondria. This compound is a very potent antioxidant at low concentrations, with an excellent 1000/1 antioxidant/pro-oxidant concentration ratio. Another interesting related compound is the fluorescent rhodamine hybrid SkQR1 (Figure 14). Both compounds have shown good neuroprotective properties in a model of middle cerebral artery occlusion, when introduced immediately after reperfusion [61]. SkQ1 has also been studied in animal models against Alzheimer’s disease [62].

### 2.3. Tocopherol-Derived Quinones

The term “vitamin E” encompasses eight naturally occurring lipophilic compounds, including four tocopherols (α-, β-, γ- and δ-tocopherol) and four tocotrienols (α-, β-, γ- and δ-tocotrienol) [63]. Tocopherols exert an antioxidant response on several reactions, most notably lipid peroxidation [64] thanks to their localization in cell membranes, where they interrupt the propagation phase of the chain reaction of lipid peroxidation induced by oxidative stress mediated by ROS [65]. 

**Figure 15:**
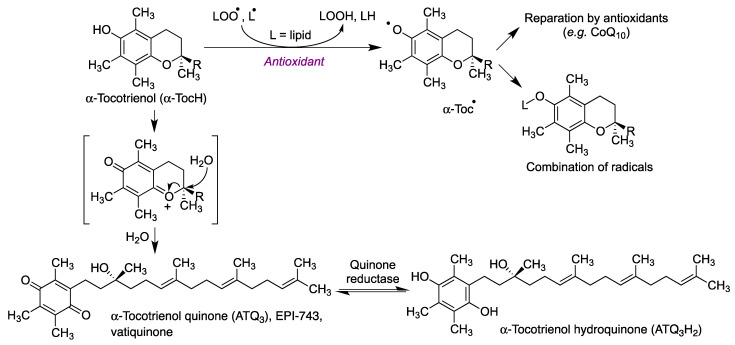
Mechanism of the antioxidant activity of tocopherol in cell membranes and its metabolism to yield α-tocotrienol quinone (vatiquinone) and its α-tocotrienol hydroquinone.

The oxidative metabolism of tocopherols leads to the formation of a quinone, presumably by oxidation to an oxonium intermediate followed by opening of the pyrane ring by reaction with a molecule of water (Figure 15) [66]. This quinone (vatiquinone, EPI-743, α-tocotrienol quinone) modulates the oxidative stress response, and its activity is dependent upon a reversible two-electron reduction to the corresponding hydroquinone (α-tocotrienol hydroquinone) [67]. Tocopherols also prevent ferroptotic cell death, a property that has been ascribed to redox modulation of 15-lipooxygenase by the corresponding hydroquinone metabolites [68]. Ferroptosis is a type of cell death caused by the excess of free intracellular iron that promotes lipid peroxidation via the formation of hydroxyl radicals by the iron-catalyzed Fenton reaction [69]. The brain is one of the organs known to be affected by iron homeostatic disruption and thus, not unexpectedly, ferroptosis is associated with some neurodegenerative diseases, including Alzheimer’s, Parkinson’s and Huntington’s diseases [68,70].

Vatiquinone protects mitochondria in vitro from oxidant insults with an extreme potency (10^3^ to 10^4^—fold over CoQ10 or idebenone). Moreover, it is orally absorbed and has a good permeation across the blood–brain barrier. It shows high promise for the treatment of Leigh syndrome and other inherited mitochondrial diseases [71], and it has also been clinically evaluated for several neurodegenerative diseases, having been granted by the FDA orphan status for the treatment of Friedrich’s ataxia [72].

### 2.4. Vitamin K and Related Quinones

Vitamin K belongs to a group of lipid-soluble compounds having a common 2-methyl-1,4-naphtoquinone ring and differing in their substitution at C-3 [73]. It is found in green leafy vegetables in the form of phylloquinone (PK, vitamin K_1_) [74,75] and is synthesized in bacteria as menaquinone (MK) [71]. Humans can synthesize menaquinone-4 (MK-4) by action of the UBIAD1 (UbiA phenyltransferase containing 1) enzyme [76]. Several reports have shown the presence of MK-4 in brain homogenates [77], being predominant in midbrain and pons medulla [78].

Vitamins K are cofactors for a carboxylase essential for the blood clotting cascade, having therefore a well-known pro-coagulant effect. Additionally, their deficiency seems to be associated to cognitive decline and they also have neuroprotective properties. Several reports have shown that vitamins K can influence the homeostasis of brain sphingolipids [69], whose alteration is linked to Alzheimer’s and Parkinson’s diseases [79,80]. It has been confirmed that in primary culture of oligodendrocyte precursors and immature fetal cortical neurons, MK-4 (and, to some extent, vitamin K_1_) prevented the depletion of glutathione mediated by free radical-mediated oxidative injury [81]. This neuroprotective profile has been confirmed by another study using methylmercury for obtaining a model of glutathione depletion [75]. Moreover, vitamin K_3_ inhibits the aggregation of the Aβ_1–42_ protein, and it is also neuroprotective against Aβ_1–42_ insult in human neuroblastoma cells (SH-SY5Y) [82].

**Figure 16:**
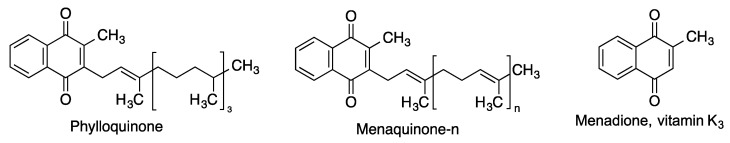
Structures of neuroprotective vitamin K-related quinones.

Menadione (vitamin K_3_, Figure 16), a simplified vitamin K analogue of synthetic origin, is a potent competitive inhibitor of monoamino oxidases (MAO), with some selectivity for MAO-B [83]. This is in itself an interesting feature in antiparkinsonian drug design because MAO-B is in charge of the metabolic degradation of dopamine. Moreover, MAO activity is associated to oxidative stress due to the formation of hydrogen peroxide in the course of the oxidative demethylation mechanism, and indeed MAO-B is elevated during aging and in several neurodegenerative disorders [84].

## 3. Non-Endogenous Quinones

### 3.1. Embelin

Embelin is a natural 1,4-benzoquinone derivative, isolated from the Indian medicinal herb *Embelia ribes* and having an acceptable oral bioavailability [85] and interesting therapeutic potential in several areas [86,87]. In the course of a project aiming at the identification of natural products as multitarget compounds against Alzheimer’s disease, this compound was found to inhibit beta-site amyloid precursor protein cleaving enzyme (beta-secretase 1, BACE-1), acetylcholinesterase and butyryl cholinesterase, with a balanced multitarget profile [88]. On the basis of kinetic studies and molecular modeling, embelin was proposed to inhibit cholinesterases via interaction with their allosteric peripheral anionic site. Embelin also enhanced the activity of the P-gp glycoprotein in LS-180 cells, via protein induction. This is an interesting feature from the point of view of Alzheimer’s disease treatment, since this efflux pump takes part in the clearance of amyloid-β from the brain of AD patients [89]. Embelin was shown to reduce cognitive deficit in a scopolamine-induced Alzheimer’s disease-like condition in a rat model [90]. Embelin is also a potent radical scavenger [91] and shows anti-inflammatory properties, which are also shared by some of its *N*-arylimines [92].

**Figure 17:**
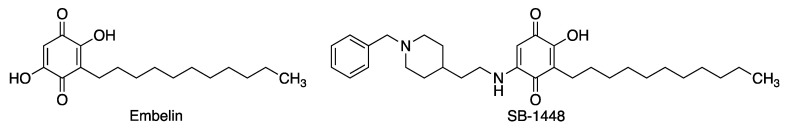
Structures of embelin and its analogue, the embelin-donepezil hybrid SB-1448.

SB-1448 (Figure 17) is an analogue of embelin designed by incorporation of a fragment related to the cholinesterase inhibitor donepezil. This compound inhibits both cholinesterases and also A*β* self-aggregation. Moreover, it has good pharmacokinetic properties, since it is orally bioavailable and crosses the blood−brain barrier. Orally administered SB-1448 reduces scopolamine-induced cognitive impairments in mouse models [93].

### 3.2. APX-3330 (E-3300)

**Figure 18:**
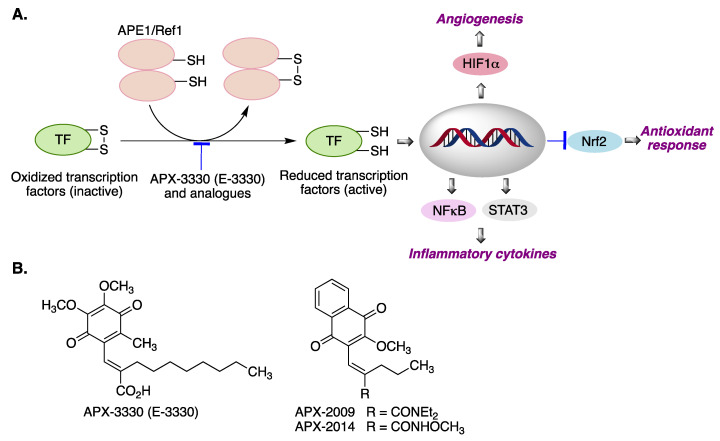
(**A**) Activation of transcription factors by the APE1/Ref-1 protein and its biological consequences. (**B**) Structures of the APE1/Ref-1 inhibitors APX-3330, APX-2009 and APX-2014. Abbreviations: APE1/Ref1: Apurinic/apyrimidinic endonuclease/redox-factor 1. TF: transcription factor. HIF-1α: Hypoxia-inducible factor 1-alpha. NF-κB: Nuclear factor kappa-light-chain-enhancer of activated B cells. STAT3: Signal transducer and activator of transcription 3. Nrf2: nuclear factor erythroid 2–related factor 2.

APE1/Ref-1 (APE1) is a multifunctional protein with both DNA repair and transcriptional regulatory activities [94]. Furthermore, it has an important role in controlling cellular response to oxidative stress and inflammation [95] APE1 expression varies in different tissues, and its expression is generally increased in the nervous system, being particularly high in the brain and spinal cord of patients with amyotrophic lateral sclerosis (ALS) and in the cerebral cortex of Alzheimer’s disease patients, as an adaptive response to oxidative stress [96]. APE1/Ref-1 activates a variety of transcription factors via disulfide bridge reduction. Relevant transcription factors (TFs) regulated by APE1/Ref-1 include HIF-1α, NF-κB and STAT3, and it is also a negative regulator of Nrf2 [97] (Figure 18A). The best-known inhibitor of the APE1/Ref-1 redox function is the benzoquinone derivative APX-3330 (E-3330), which is undergoing clinical trials for the treatment of diabetic retinopathy [94]. Some related compounds derived from naphthoquinone include APX-2009 and APX-2014, which are promising for targeting neovascular eye diseases [98] (Figure 18B).

### 3.3. Cannabinoid-Derived Quinones

**Figure 19:**
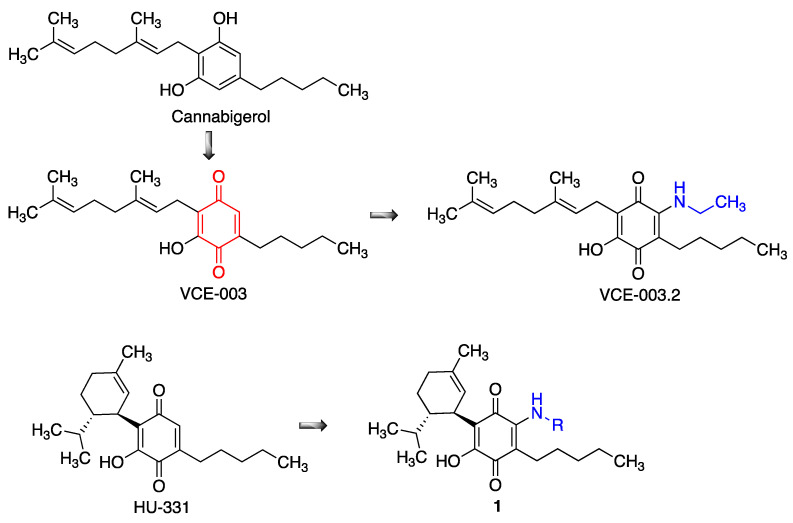
Stages in the design of neuroprotective cannabinoid-derived quinones.

Cannabinoids promote neural progenitor cell proliferation, regulate neural cell differentiation and exert neuroprotective actions in several models of neurodegenerative diseases. These effects are generally accepted to be mostly mediated via the presynaptic CB1 receptor, but unfortunately their activation also results in a psychoactive response. However, some non-psychoactive cannabinoids such as cannabigerol (CBG), which does not bind to CB1 receptors, exert neuroprotection via PPARγ activation, which is also known promote neural repair in neurodegenerative conditions. The quinone derivative VCE-003 is more potent as a the PPARγ activator, and was found to alleviate neuroinflammation in a chronic model of multiple sclerosis [99]. However, this compound is also a potent immunosuppressor, which may be due to the formation of covalent adducts by addition of the thiol group in cysteine residues to the electrophilic quinone moiety [100]. For this reason, an additional ethylamino substituent was added to the only free position of VCE-003, furnishing the non-thiophilic quinone VCE-003.2 (Figure 19). This compound was shown to enhance neuronal progenitor cell survival and to also provide a symptomatic relief in murine models of Huntington’s disease [101], and it also showed protective effects in SOD1G93A transgenic mice, a model of amyotrophic lateral sclerosis [102]. Interestingly, other cannabinoid-derived *p*-quinones, such as HU-331, have shown good anticancer properties [103], besides having dual activity as Nrf2 activators and BACH1 inhibitors [104]. Related quinones **1**, structurally related to menthol, have also been studied for neuroprotective activity [105].

### 3.4. Asterriquinones and Other Indolylquinones

Asterriquinones are naturally occurring bis-indolyl-dihydroxy- or dimethoxybenzoquinones isolated from several fungal species and strains including *Aspergillus terreus*, from which their name arises. Asterriquinone B4 has shown neuroprotective activity in several cellular models using oxidative stress-inducing neurotoxics mainly connected to Parkinson’s disease such as rotenone and 6-hydroxydopamine (6-OHDA) [106].

**Figure 20:**
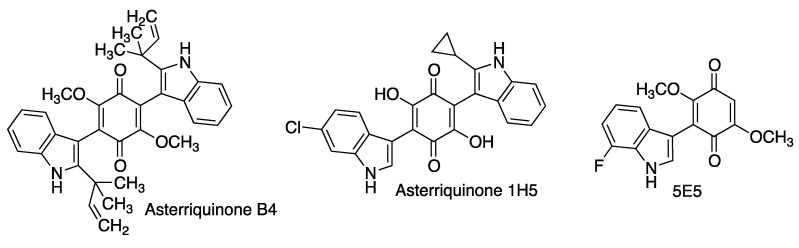
Neuroprotective asterriquinones and simpler indolylquinones.

Neurotrophins are members of the nerve growth factor (NGF) family of proteins that induce the survival and development of neurons and therefore their activation can be an interesting approach to the treatment of neurodegenerative diseases. In the course of a project aiming at the discovery of small-molecule NGF activators with good cell- and blood-brain-barrier permeability, combinatorial libraries of asterriquinones and mono-indolylquinones were screened in a test able to detect phosphorylated TrkA, the NGF receptor. Some of these compounds showed good activity coupled with low toxicity, most notably asterriquinone 1H5 and especially the mono indolylquinone 5E5, a very potent activator of TrkA at non-toxic concentrations (Figure 20). In combination with a sub-therapeutic dose of nerve growth factor, this compound was shown to promote neuronal differentiation of PC12 cells [107]. 

### 3.5. Pyrroloquinolinequinone (PQQ)

**Figure 21:**
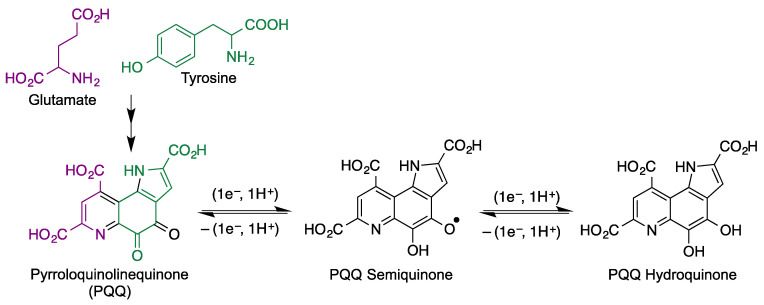
Biosynthetic origin of PQQ and its redox equilibria with PQQ semiquinone and hydroquinone.

Quinoproteins [108] are a class of oxidoreductases that have as a cofactor an amino acid-derived *ortho*-quinone moiety, including topaquinone (TPQ, 2,4,5–trihydroxyphenylalanine quinone), lysine tyrosylquinone (LTQ), cysteine tryptophylquinone (CTQ) and tryptophan phyloquinone (TTQ), and, especially, pyrroloquinolinequinone (PQQ). Unlike the other members of the family, PQQ is bound non-covalently to its proteins, which are redox enzymes in the mitochondrial respiratory chains. Despite being biosynthesized in bacteria (from glutamate and tyrosine), it has been found in rats and humans [109,110] due to its presence in commonly consumed foods such as vegetables and meats [111,112]. PQQ is capable of continuous redox cycling (Figure 21) and is a very potent radical scavenger that is active at picomolar levels and is at least 100 times more efficient than ascorbic acid, menadione, isoflavonoids and polyphenolic compounds in terms of redox cycling potential [113,114]. Besides its high activity as a radical scavenger, PQQ is able to reduce oxidative stress by increasing the expression of Nrf2 [115].

**Figure 22:**
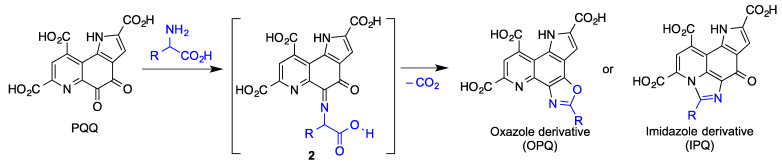
Products derived from the reaction of PQQ with amino acids such as free glutamate in the brain.

In an artificial aging model induced with D-galactose, PQQ was able to reduce oxidative stress, as measured by the generation of malondialdehyde (MDA), and to increase the activity of superoxide dismutase (SOD), the most important antioxidant enzyme serving as a defense against ROS. Also, PQQ inhibited excitotoxicity due to its ability to bind covalently to free glutamate in the brain giving intermediate imine **2** and then fused oxazole or imidazole derivatives, as shown in Figure 22 [116]. The protection exerted by PQQ in primary cultured hippocampal neurons against glutamate-induced cell damage has been ascribed to activation of phosphatidylinositol 3-kinase/protein kinase B (PI3K/Akt) signaling through modulation of glutamate-induced imbalance between B-cell lymphoma 2 (Bcl-2) and Bcl-2-like protein 4 (Bax). Finally, PQQ reduced the activity of GSK-3β via the PI3K/Akt pathway resulting in a down-regulation of p-Tau at the hippocampus, which is instrumental for learning and memory processes in aging [117].

In an experimental model of Parkinson’s disease (PD), PQQ exerted effective protection in SH-SY5Y cells against 6-hydroxydopamine-induced neurotoxicity, playing a critical role as an antioxidant [113,114]. In a rotenone model of PD, PQQ showed protective effects both in vitro and in vivo, being able to reduce oxidative stress and improve mitochondrial functions and dopamine redistribution [118].

### 3.6. Geldanamycin and Its Analogues

The heat shock protein Hsp90 is a chaperone required for the proper folding and functionality of a variety of proteins. Geldanamycin is a natural antibacterial, antifungal and anticancer benzoquinone ansamycin that binds to Hsp90 and enhances degradation of its client proteins, and is gaining recognition as an emerging target in neurodegeneration [119,120,121]. Thus, the levels of Hsp90 are increased in the brains of Parkinson’s disease patients and correlates well with increased levels of insoluble α-synuclein, whose accumulation leads to dopaminergic neuronal death and hence to dopamine depletion [122]. Indeed, geldanamycin has been shown to protect against MPTP-induced dopaminergic neurotoxicity [123], and also to rescue striatal dopamine levels but not α-synuclein-induced neuronal loss [124]. Geldanamycin has been effective in several preclinical studies of Alzheimer’s disease, showing the ability to suppress memory deficits in β-amyloid-injected rats [125] and induce the degradation of misfolded tau protein [126] and has also been shown to reduce brain injury in a mouse model of intracerebral hemorrhage and improve the neurological outcome of these mice [127].

Tanespimycin (17-allylamino-17-demethoxygeldanamycin, 17-AAG) is another potent inhibitor of Hsp90 that attenuates Aβ-induced synaptic toxicity and memory impairment [128]. Alvespimycin (17-DMAG), having a (dimethylaminoethylamino) substituent at C-17 instead of a methoxy, is a water-soluble geldanamycin derivative that potently inhibits Hsp90 and is also anti-inflammatory by interfering with NF-κB signaling. This compound reduces polyglutamine-mediated motor neuron degeneration through proteasomal degradation of the androgen receptor in a mouse model [129]. It has also been shown to improve balance and coordination in a mouse model of the Machado-Joseph disease (spinocerebellar ataxia type 3), a neurodegenerative disease for which there is no treatment [130].

**Figure 23:**
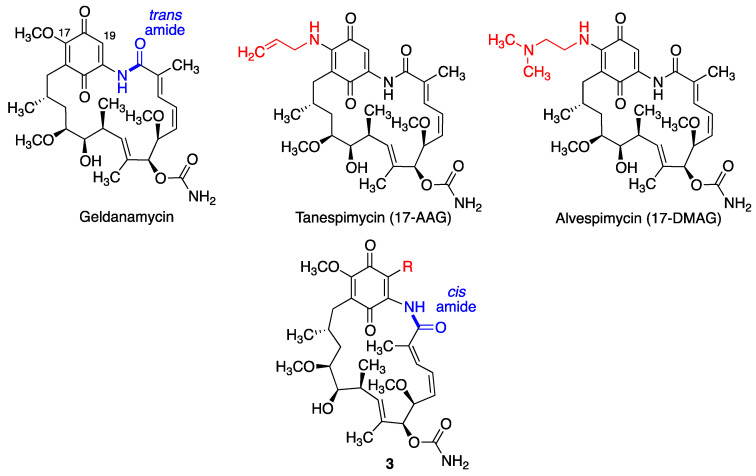
Geldanamycin and its neuroprotective analogues.

In spite of its interesting properties, geldanamycin shows an unacceptable hepatotoxicity that has been proposed to arise from the Michael addition of biological nucleophiles to its unsubstituted 19-position. Due to the electron-releasing effect of the allylamino group, the electrophilicity of tanespimycin and alvespimycin is decreased, and their toxicity is accordingly lower. In an alternative approach, C-19 substituted derivatives of geldanamycin were designed in order to increase its stability. Interestingly, the introduction of alkyl and aryl substituents at C19 (compounds **3**, Figure 23) resulted in *trans* to *cis* isomerism of the lactam group as a result of steric strain and therefore in a conformational change in the macrocyclic ring. These derivatives maintained the ability to inhibit Hsp90 while showing much lower toxicities [131].

### 3.7. Rifampicin and Its Quinone

**Figure 24:**
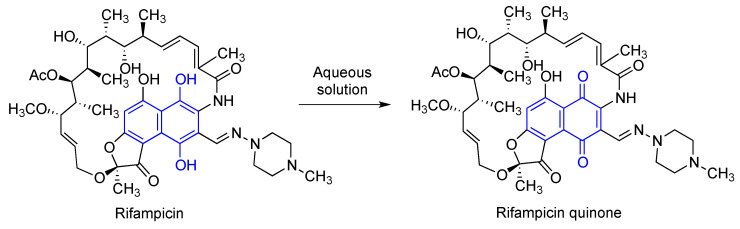
Structures of rifampicin and its quinone.

Rifampicin (Rif) is a natural macrocyclic bactericidal antibiotic, broadly used for the treatment of *Mycobacterium* infections, including leprosy and tuberculosis [132,133]. In aqueous solution, Rif is spontaneously oxidized to rifampicin quinone (RifQ) [134], as shown in Figure 24. Rifampicin was the first well-established antibiotic having therapeutic effects in chronic neurodegenerative disorders, which were observed in patients with leprosy on Rif chronic treatment, who had a lower prevalence of dementia [135].

It has been stablished that Rif and RifQ inhibit key markers of inflammation, such as TNF-α and IL-6 and control oxidative stress associated to PI3K inhibition. They both diminished the activation of primary microglial cells induced by α-synuclein fibrillary aggregates and RifQ also ameliorated the neurotoxic effects mediated by these fibrils in microglial cells [136]. RifQ was also observed to exert more potent anti-inflammatory effects than Rif [137]. An immunosuppressive effect of RifQ has also been observed in microglial cells [137,138]. In another study, it was shown that Rif and RifQ protected PC12 cells against apoptosis induced by *N*-methyl-4-phenylpyridinium (MPP^+^) and suppressed expression of α-synuclein multimers, being more potent the oxidized form of Rif [139].

In additional experiments, oral Rif has shown improvement in memory and on amyloid pathology in the β-amyloid oligomer mice model by reducing β-amyloid oligomers and tau hyperphosphorylation [140]. Another study confirmed that Rif inhibited β-amyloid aggregation and neurotoxicity in a concentration-dependant manner. By studying the antiaggregating activity of several rifampicin analogues, the authors concluded that the inhibitory activities of rifampicin against β-amyloid aggregation and neurotoxicity are associated to its hydroquinone moiety, due to its activity as a radical scavenger [141]. On the other hand, some experiments have demonstrated that RifQ may be the most active species in inhibiting fibrillation and disaggregating of formed fibrils [134].

### 3.8. Miscellaneous Carbocyclic Quinones

**Figure 25:**
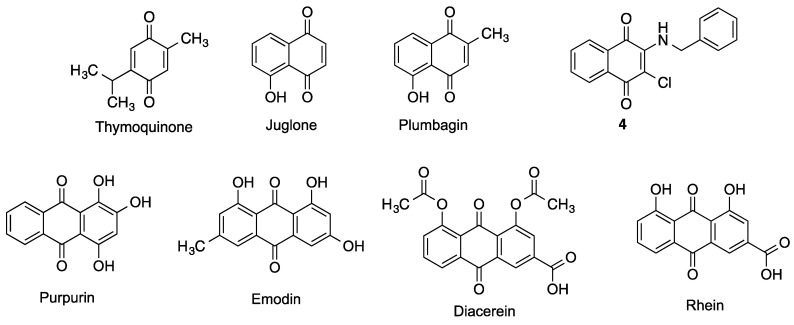
Miscellaneous neuroprotective benzoquinones, naphthoquinones and anthraquinones.

Several additional structurally simple carbocyclic quinones, summarized in Figure 25, will be discussed in this Section.

#### 3.8.1. Benzoquinones

Thymoquinone is a natural product isolated from the seeds of *Nigella sativa* that crosses the blood brain barrier and shows potent antioxidant and anti-inflammatory properties. Thymoquinone provides protection against ischemic brain damage and reduces oxidative damage associated to epileptic seizures and exposure to several toxins and ionizing radiation [142].

#### 3.8.2. Naphthoquinones

Juglone is an allelopathic 1,4-naphthoquinone derivative produced by several plants of the *Juglandaceae* family, being particularly abundant in black walnuts (*Juglans nigra*). It has been studied as a cytotoxic agent for anticancer therapies, but also some interesting neuroprotective effects have been described. Juglone was found to inhibit BACE and Aβ aggregation and induce Aβ fibril disaggregation, with comparable potencies on all the three targets. Modifications on the naphthoquinone core with different substituents led in almost all cases to reduction or even loss of these activities [143]. Juglone has also been shown to be an efficient inhibitor of peptidyl-prolyl *cis*/*trans* isomerase Pin1, a rotamase involved in the regulation of the phosphorylation status of Tau protein at Thr-231, a key residue for the interaction with microtubules [144]. Thus, juglone was able to partially reverse the Thr-231 dephosphorylation of Tau induced by oxidative or heat stress in primary cortical cultures, although the implications of these results are still to be fully defined [145]. These observations, taken together with other evidence of juglone as a redox cycling [146] and chelating agent [147] confer to this molecule a high neuroprotective potential.

Plumbagin is another natural compound with a 1,4-naphthoquinone core, isolated from plants of several genera such as *Plumbago*, *Drosera* and *Nepenthes*, and endowed also with an allelopathic function. This phytochemical is attracting increasing interest since it was established as a Nrf2-pathway inducer, having shown the ability to protect neurons against oxygen-glucose deprivation as a model of ischemia, and also reducing the extent of brain damage and neurological deficit associated to middle cerebral artery occlusion/reperfusion in mice [148]. Plumbagin also prevented the cognitive decline of mice treated with streptozotocin, a toxin that leads to an AD-like condition. The neuroprotective effect of plumbagin in this model was ascribed to the activation of Nrf2-ARE pathway, the inhibition of BACE-1 and an attenuation of astrocyte hyperactivation [149]. Other mechanisms may be involved in the global neuroprotective effect of plumbagin, as it has been identified also as a NF-kB pathway inhibitor [150] and it displays antioxidant and chelating activities [151].

Two libraries of naphthoquinone and anthraquinone derivatives bearing arylamino chains were investigated for their in vitro activity against a number of targets relevant to Alzheimer’s disease. The naphthoquinone derivative **4** showed the best multitarget profile, with good potency as an inhibitor of the aggregation of Aβ_40_ and the PHF6 tau fragment and inhibition of acetylcholinesterase (AChE) and MAO B. This compound also impaired Aβ_42_ fibril formation and elicited some neuroprotection against Aβ_42_ toxicity in primary cultures of cerebellar granule cells [152].

#### 3.8.3. Anthraquinones

Several natural phenolic compounds derived from an anthraquinone framework have shown neuroprotective potential. Purpurin (1,2,4-trihydroxyanthraquinone) is a natural dye extracted from the roots of madder plants (*Rubia tinctorum*, *Rubia cordifolia*) that exerts a variety of neuromodulatory effects. Purpurin shows a promising profile to address Tau pathologies, since it was able not only to hamper the aggregation of PHF-6 (highly aggregation-prone hexapeptide fragment, a simplified model of Tau), but also to diassamble pre-formed PHF-6 fibrils. These effects could be attributed to its ability to establish hydrophobic contacts with PHF6 and full-length Tau, in a dose-dependent manner. Moreover, purpurin relieved neurological symptoms of transgenic *Drosophila* flies overexpressing human Tau protein by reducing its phosphorylation and accumulation, as well as it did in neuroblastoma cultured cells overexpressing Tau. Moreover, purpurin is able to cross the blood-brain barrier [153]. It also shows interesting antioxidant and anti-inflammatory profiles, confirmed by its ability to protect HT22 cells from oxidative stress induced by H_2_O_2_ and the mitigation of locomotor impairment, microglia activation and neuronal death in gerbils suffering from transient forebrain ischemia. All these observations were ascribed to the modulation of several antioxidant signaling pathways and the inhibiton of NF-κB activation [154]. The same group tested purpurin in an aging mouse model induced by D-galactose, observing a significant decrease in oxidative stress and inflammation markers, as well as an alleviation of memory impairment and reduction of proliferating neuroblasts in the subgranular zone of the dentate gyrus [155]. These findings promote purpurin as a promising antineurodegenerative agent.

Emodin occurs in several plants used in the traditional Chinese medicine, but also in certain lichens and fungi. It displays a number of pharmacological activities such as laxative or antibacterial, but also its anti-inflammatory and antioxidant profile have attracted some interest to target neurodegeneration. In particular, emodin has been found to be a good radical scavenger and chelating agent, a Nrf2 inducer and an efficient inhibitor of Aβ_42_ and Tau aggregation and toxicity, as well as it inhibits some enzymes related to neurodegenerative processes, such as AChE or BACE [156]. Some in vivo studies also confirm the capacity of emodin to reduce cognitive impairment, neuroinflammation, senile plaques formation and neuronal death in several rodent models of neurodegenerative diseases [157]. Even if there are some safety concerns, mainly related to nephrotoxicity and hepatotoxicity, it seems that toxic doses are in general much higher than those showing therapeutic effects, so emodin maintains its potential to take part in drug design efforts against neurodegenerative diseases.

There are some other anthraquinones obtainable from plants of the *Rheum* genus (rhubarb) with relevant pharmacological activities, from which we will highlight rhein, a well-known anti-inflammatory compound. Its prodrug diacerein is approved for the treatment of some inflammatory diseases such as rheumatoid arthritis. Rhein has been studied for the treatment of traumatic brain injury (TBI), showing a good permeability through the blood-brain barrier and displaying a significant protection of this barrier after a TBI event in rats [158]. This activity seems to arise from NADPH oxidase inhibition, avoiding the increase of ROS levels that subsequently activates the ERK/MMP-9 pathway, thus limiting changes in the permeability and disruption of BBB [159]. Moreover, rhein attenuated inflammation markers, pyroptosis and neurological dysfunction in mice subjected to TBI [160]. Modulation of inflammatory response could be exerted by the NF-kB-pathway and NLRP-3 inflammasome inhibition [161], although additional mechanisms can also contribute to the observed activity. In addition, rhein is a moderate inhibitor of AChE (IC_50_ = 18.1 ± 0.24 µM), a potency that is not interesting in itself, but that is suitable for the design of multitarget ligand with balanced activities, as will be discussed below (Section 4.3).

### 3.9. Miscellaneous Heterocyclic Quinones

**Figure 26:**
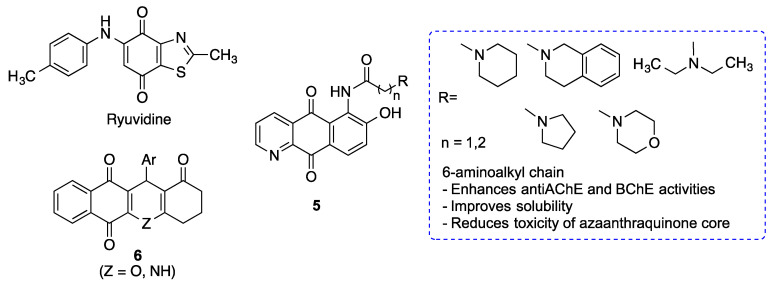
Structures of some neuroprotective heterocyclic quinones.

The TDP-43 protein (TAR DNA binding protein 43) accumulates in the cytoplasm of neurons in patients with amyotrophic lateral sclerosis (ALS) and frontotemporal dementia (FTLD), which led to the classification of many ALS and FTLD cases as proteinopathies. For this reason, TDP-43 can be considered a key therapeutic target for the treatment of these and perhaps other neurodegenerative diseases [162]. Screening experiments have shown that kinase inhibitors, particularly inhibitors of cyclin-dependent kinases (CDKs) and glycogen synthase kinase 3 (GSK3), modulate TDP-43 accumulation. More specifically, the heterocyclic quinone ryuvidine, a CDK inhibitor (Figure 26), inhibited TDP-43-positive stress granule formation, although the detailed mechanism of this process was not identified [163].

A family of 6-substituted aza-anthraquinones was designed taking into account the pharmacological activities previously described for 9,10-anthraquinones and alkaloids with an aza-anthraquinone core, particularly those related to Alzheimer´s disease: inhibition of AChE, BChE (butyrylcholinesterase) and Aβ fibril formation and deposition. Alkylamino chains were introduced on the position 6 to enhance anti-neurodegenerative activities, but also to avoid the drawbacks of many of the original structures, such as poor solubility or toxicity. The resulting derivatives (compounds **5**) showed low toxicity on immortalized cultures of neuroblastoma cells and macrophages, and a significant neuroprotective activity against H_2_O_2_-induced oxidative stress. Furthermore, they were able to reduce the levels of inflammatory cytokines and NO produced by macrophages exposed to LPS, and also the production and aggregation of Aβ. Moderate AChE and BChE inhibitory activites were also found, with a slight selectivity for the former. Compounds **5** showed, in general, a promising multitarget profile to address Alzheimer´s disease [164].

Polyheterocyclic quinones **6** have been identified as inhibitors of the JAK-STAT (Janus Kinase-Signal Transduction) pathway, which is of relevance to immunological and inflammatory processes [165]. The JAK–STAT3 pathway is being increasingly recognized as a regulator of astrocytic activation and glial scar formation in damaged brain regions and thus the modulation of STAT3 signaling may help to control the excessive gliogenic environment and enhance neural repair [166]. Moreover, neuroinflammatory diseases such as Parkinson’s disease are characterized by an aberrant activation of the JAK/STAT pathway and can therefore be potentially treated with JAK/STAT inhibitors [167]. Regarding Alzheimer’s disease, STAT3 activation contributes to neuronal death after Aβ exposure [168] and upregulates OCIAD1 (ovarian carcinoma immunoreactive antigen domain-containing protein 1), which induces mitochondrial dysfunction and synaptic damages, thereby contributing to neurodegeneration [169].

## 4. Hybrid Compounds Based on Quinone Moieties

The multitarget ligand approach seems to be particularly well suited for drug discovery in complex, multifactorial diseases such as neurodegenerative diseases [170,171,172]. The simplest approach to compound design in this area is the combination of structural fragments known to bind two or more targets that are validated to combat the disease, leading to hybrid structures. 

### 4.1. Memoquin

**Figure 27:**
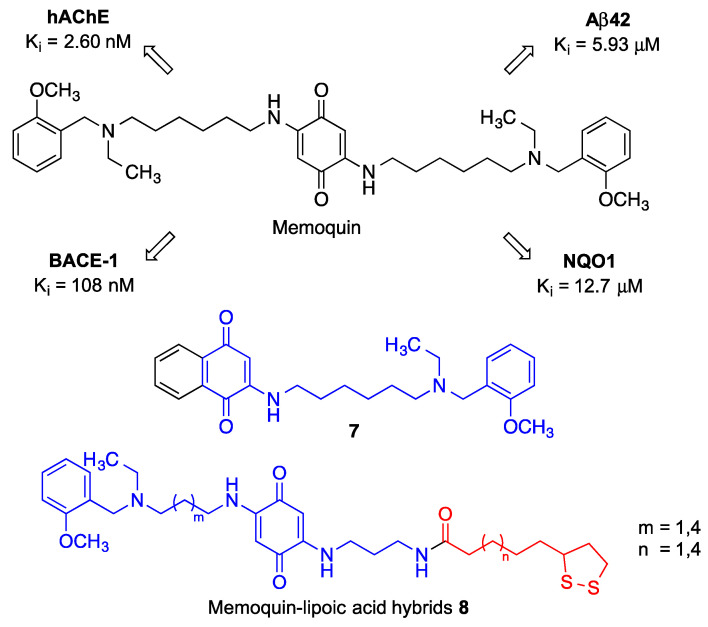
Structure and pharmacological profile of memoquin. Structures of a half-molecule simplified memoquin analogue **7** and memoquin-lipoic acid hybrids **8**.

Memoquin, designed as a hybrid between benzoquinone and polyamines, which are known to prevent cognitive impairment [173]. Memoquin can be regarded as the pioneering example of the application of the multitarget approach against neurodegenerative diseases. Thus, memoquin shows a broad spectrum of activities, including ROS scavenging and inhibition of β-amyloid aggregation, BACE-1 activity and acetylcholinesterase. The antioxidant properties of memoquin were evaluated by direct ROS scavenging and via its prior transformation into its hydroquinone derivative by the NAD(P)H quinone oxidoreductase 1 (NQO1). This hydroquinone may scavenge ROS to afford again the initial quinone, which is able to re-start the redox cycle. It is important to note that NQO1 is overexpressed in hippocampal neurons in AD patients. Furthermore, memoquin inhibits AChE and BACE-1 at nanomolar concentrations, thus being able to improve the survival of cholinergic neurons and decrease tau hyperphosphorylation. All these pharmacological activities (Figure 27), coupled to its ability to cross the blood-brain barrier, explain the ability of memoquin to perform memory improvements in several animal models of dementia [174].

In an effort to reduce the molecular weight of memoquin while preserving its multitarget profile, a library of monosubstituted naphthoquinones bearing a single side chain was developed. In contrast with other analogues modified at the terminal tertiary amine function, Compound **7**, which can be viewed as half memoquin, retained the cholinesterase inhibitory activity, which underscores the importance of the ethyl and 2-methoxybenzyl moieties. Compound **7** also showed β secretase inhibitory activity but, on the other hand, its potency as an inhibitor of self-induced Aβ aggregation was low [175].

A small library of memoquin-lipoic acid hybrids was also synthesized, aiming at enhancing the antioxidant profile through radical scavenging by lipoic acid. One of the terminal 2-methoxybenzyl functions of memoquin was replaced by a lipoic acid unit, joining both pharmacophores by polyamine linkers to furnish lipoamides **8**. These derivatives displayed a significant capacity to inhibit ROS production and ROS-induced cytotoxicity, which, interestingly, were studied in cells treated with sulforaphane, a Nrf2 inducer that increases the levels of the NQO1 enzyme [176]. This experimental design was based on the fact that the hydroquinones resulting from NQO1 reduction of **8** are the actual antioxidant species. Compound **8** were also able to reduce Aβ_42_ self-aggregation and retained a moderate AChE and BChE inhibitory activity, more selective for the former but lower than memoquin in both cases, although it is still valuable taking into account that, in multitarget drug design, an equilibrated profile is more desirable than very potent but unbalanced activities [177].

**Figure 28:**
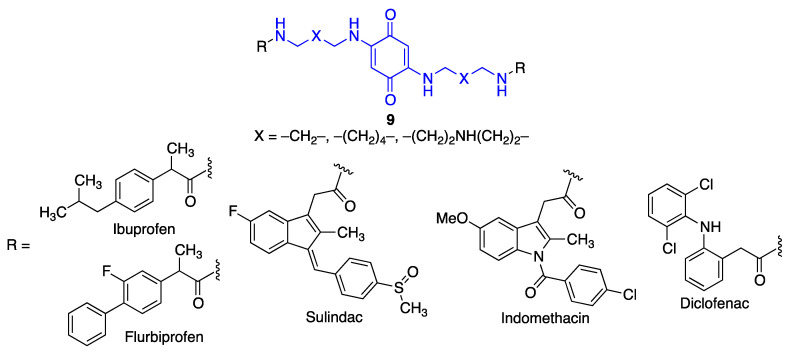
Memoquin analogues that include fragments corresponding to non-steroidal anti-inflammatory drugs.

Another small library of hybrid compounds (**9**) was designed, comprising a benzoquinone core and a structural fragment corresponding to various non-steroidal anti-inflammatory drugs. These moieties were connected through polyamine linkers, leading to analogy with memoquin (Figure 28). These compounds retained or even increased the potent anti-aggregating activity of the parent quinone, while exhibiting AChE inhibitory activities in the µM range [178].

### 4.2. Quinone-Cholinesterase Inhibitor Hybrids

**Figure 29:**
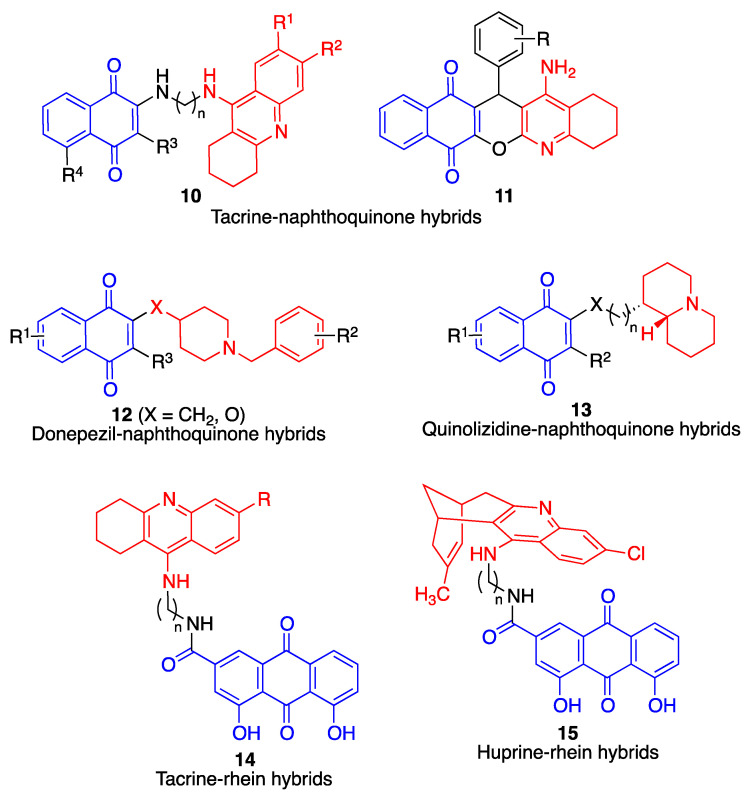
Multitarget hybrid compounds with cholinesterase inhibitory activity.

The most common proteins targeted by potential anti-AD multitarget compounds are cholinesterases, β-amyloid and tau. Figure 29 summarizes the main families of hybrid compounds designed on these premises and containing quinone moieties. 

Compounds **10**, containing structural fragments corresponding to naphthoquinone (including the natural product juglone) and tacrine, linked by a spacer, showed potent activity as acetylcholinesterase inhibitors, inhibition of spontaneous amyloid aggregation, and a low toxicity in immortalized mouse cortical neurons Neuro2A and primary rat cerebellar granule neurons [179]. The compounds also displayed antioxidant activity, which, as discussed above for the case of compounds **8**, was studied in conditions that stimulated the activity of NQO1 in order to generate the active hydroquinone species.

Compounds **11**, described by the authors as quinopyranotacrines and containing juxtaposed tacrine and naphthoquinone moieties, were less toxic than tacrine, induced acetylcholinesterase inhibition with moderate activity and were radical scavengers showing up to 3.74 trolox equivalents in the ORAC assay, although they were not studied for their ability to inhibit β-amyloid aggregation and deposition [180]. 

A library of merged donepezil-naphthoquinone derivatives (compounds **12**) showed a potent and selective butyrylcholinesterase inhibitory activity, together with antioxidant and amyloid antiaggregating properties. These compounds were designed to have lower molecular weight, and therefore an improved pharmacokinetic profile, over hybrid structures arising from joining two active compounds with a spacer chain [181].

The multitarget profile of a library of compounds combining naphthoquinone and quinolizidine frameworks (compounds **13**) was studied. These compounds displayed inhibitory activity on both AChE and BChE, with preference for the former, but showed modest activity on the spontaneous aggregation of β-amyloid [182]. Because docking studies suggested interactions with the cholinesterase peripheric anionic site (PAS), the ability of the compounds to inhibit AChE-induced amyloid fibrillation, arising from interaction of amyloid protein with some hydrophobic residues of PAS, was studied with positive results for all compounds **13**. 

Tacrine has also been hybridized with rhein, a natural anthraquinone derivative present in rhubarb, leading to compounds **14**. In comparison with tacrine, these hybrids showed an enhanced inhibition of AChE and a reduced ability to block BChE activity [183].

Huprine is a synthetic compound developed by merging the structures of tacrine and the natural cholinesterase inhibitor huperzine A. It is an extremely potent (0.026 nM), reversible inhibitor of acetylcholinesterase [184]. This compound was connected via suitable spacer chains to rhein. The resulting hybrids (compounds **15**) showed good brain permeability and were found to be potent inhibitors of acetylcholinesterase, butyrylcholinesterase and BACE-1. They also displayed dual Aβ42 and tau antiaggregating activity. In vivo studies of selected compounds in APP-PS1 transgenic mice confirmed that they were able to lower the brain levels of soluble Aβ [185]. A second generation of hybrids was developed by replacing the chlorobenzene ring of the tacrine fragment by aromatic or heteroaromatic rings, but this resulted in a loss of activity in AChE and BACE-1 enzymes, even though the activity as Aβ42 and tau antiaggregating agents was retained [186].

### 4.3. Quinone-Amino Acid Hybrids

**Figure 30:**
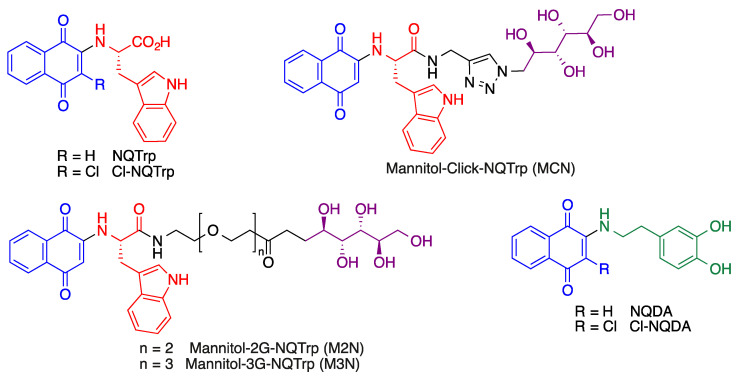
Selected naphthoquinone-tryptophan and naphthoquinone-dopamine hybrids.

Some quinone-amino acid hybrids have shown interesting properties in terms of the potential treatment of Alzheimer’s disease. The main example are the derivatives of the 1,4-naphtoquinone-tryptophan hybrid scaffold, showing protein aggregation inhibitory activity by inhibiting the process of amyloid formation and also disassembling pre-formed fibrils. These properties are associated to the ability of these quinones to generate hydrogen bonding and hydrophobic interactions with the residues responsible for the initial nucleation of protein/peptide aggregates [187]. Some representative naphthoquinone-tryptophan hybrids are shown in Figure 30.

These compounds have been studied in several models of neurodegenerative diseases. For instance, the compound NQTrp prolonged the life span and abolished the defective locomotion of Alzheimer’s disease model of *Drosophila* flies, whose brains showed a significant reduction in the accumulation of oligomeric Aβ species [188]. Similarly, NQTrp and several of its analogues inhibit the aggregation of α-synuclein (α-Syn) in vitro and reduce α-Syn-induced cytotoxicity. These findings are highly relevant taking into account that the accumulation and aggregation of this protein into amyloid fibrils is the hallmark of several neurodegenerative disorders (collectively known as synucleinopathies), most notably Parkinson’s disease. 

More recently, naphthoquinone-dopamine hybrids NQDA and Cl-NQDA have been shown to inhibit α-synuclein aggregation, disrupt preformed fibrils and reduce aggregate-induced toxicity [189]. 

## 5. Quinone Structural Fragments in the Design of Neuroprotective Prodrugs

**Figure 31:**
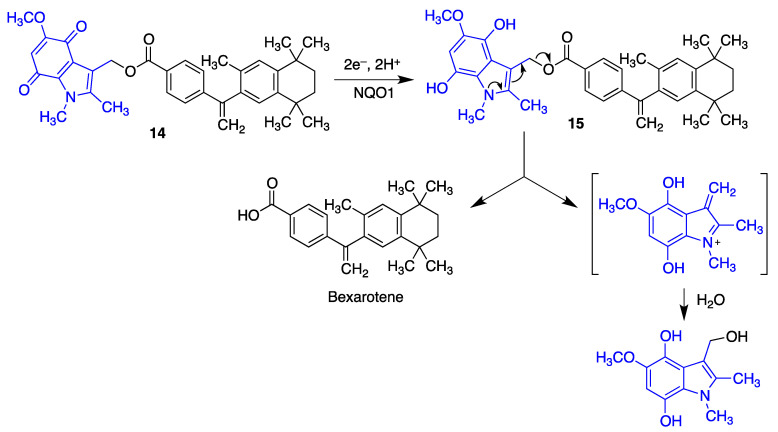
An indolequinone derivative acting as a bexarotene prodrug by a process initiated by NQO1 reduction of the quinone moiety.

Because the brain levels of NAD(P)H/quinone oxidoreductase (NQO1) are elevated in neurodegenerative diseases, it was reasoned that suitably designed quinones could be useful for specifically delivering neuroprotective compounds to their targets, avoiding systemic exposure. Bexarotene, an agonist of the retinoid X receptor (RXR), has shown neuroprotective properties in rodent models of Alzheimer’s [190] and Parkinson’s [191] diseases, amyotrophic lateral sclerosis [192] and also following traumatic brain injury [193]. However, peripheral exposure to this drug leads to side effects such as hypertriglyceridemia, hypothyroidism and leukopenia. A small library of indolequinone derivatives of bexarotene such as **14** was designed to minimize this problem. These compounds were metabolically stable in their quinone form but, as shown in Figure 31, their reduction by NQO1 affords an intermediate **15** where the increased electron density of the indole ring facilitates the extrusion of the bexarotene molecule [194].

## 6. Summary

The quinones described in this review and their neuroprotective properties are summarized in Table 1.

## 7. Conclusions

Although quinones can be either cytoprotective or cytotoxic, depending on several factors, low doses of moderately electrophilic quinones are generally cytoprotective. This is mainly due to their ability to activate the Keap1/Nrf2 pathway, which is the master regulator of the phase II antioxidant response by promoting the transcription of many enzymes involved in antioxidant and anti-inflammatory responses. Many natural and unnatural quinones, including coenzyme Q_10_, idobenone, mitoquinone, plastoquinone, vatiquinone, vitamins K, embelin, APX-3330, cannabinoid-derived quinones, asterriquinones, pyrroloquinolinequinone, geldanamycin, rifampicin quinone and memoquin, together with their analogues, show neuroprotective effects potentially relevant to the treatment of many neurodegenerative diseases. Some of them have been granted FDA orphan status for the treatment of certain neurodegenerative diseases (e.g., vatiquinone for Friedrich’s ataxia). We hope that this review will help to shed light on some misconceptions on the use of quinones in therapeutics and stimulate research into this promising area.

## Figures and Tables

**Table 1:** A summary of the quinones described in this review.

Quinone	Properties	Refs.
Coenzyme Q_10_	ROS scavenger in the mitochondria, Nrf2 inductor [38,39]. Anti-inflammatory effects, decreasing the protein and mRNA levels of pro-inflammatory cytokines. Anti-apoptotic properties [40]. Increases the levels of tyrosine hydroxylase [45].	[38,39,40,45]
QS-10	Activation of the Keap1/Nrf2 pathway. Activation of the heat-shock transcription factor-1 (HSF-1).	[51]
Idebenone	Treatment for complex I-related diseases: Leber’s hereditary optic neuropathy (LHON) Leigh syndrome, mitochondrial encephalomyopathy, lactic acidosis and stroke-like episodes (MELAS), Duchene muscular dystrophy and glaucoma. Under clinical assay for Friedreich ataxia (FRDA) [55], multiple sclerosis [56] and Parkinson’s disease [57].	[53,54,55,56,57]
Mitoquinone	Treatment of Parkinson’s disease [58] and multiple sclerosis [59].	[58,59]
SkQ1	Neuroprotective properties in a model of middle cerebral artery occlusion, when introduced immediately after reperfusion [61]. Neuroprotective properties in models of Alzheimer’s disease [62].	[61,62]
Vatiquinone (EPI-743, α-tocotrienol quinone)	Modulates the oxidative stress response [67]. Prevents ferroptotic cell death in some neurodegenerative diseases, including Alzheimer’s, Parkinson’s and Huntington’s diseases [68,70] Treatment of Leigh syndrome and other inherited mitochondrial diseases [71]. FDA orphan drug status for the treatment of Friedrich’s ataxia [72].	[67,68,69,70,71,72]
MK-4	Prevents the depletion of glutathione mediated by free radical-mediated oxidative injury [81].	[81]
Menadione (vitamin K_3_)	Protects neurons from methylmercury-induced cell death [75]. Inhibits the aggregation of the Aβ_1–42_ protein, and it is also neuroprotective against Aβ_1–42_ insult in human neuroblastoma cells (SH-SY5Y) [82]. Competitive inhibitor of monoamino oxidases (MAO), with some selectivity for MAO-B [83].	[75,82,83]
Embelin	Inhibits beta-secretase 1 (BACE-1), acetylcholinesterase and butyryl cholinesterase, with a balanced multitarget profile [88]. Reduces cognitive deficit in a scopolamine-induced Alzheimer’s disease-like condition in a rat model [90]. Potent radical scavenger [91]. Anti-inflammatory properties [92].	[88,90,91,92]
SB-1448	Inhibits acetylcholinesterase and butyryl cholinesterase [93]. Inhibits Aβ self-aggregation [93]. Reduces scopolamine-induced cognitive impairments in mouse models [93].	[93]
APX-3330 (E-3330)	Treatment of diabetic retinopathy by APE1/Ref-1 inhibition [98].	[98]
APX-2009 APX-2014	Targets neovascular eye diseases by APE1/Ref-1 inhibition [98].	[98]
VCE-003	Alleviates neuroinflammation in a chronic model of multiple sclerosis by PPARγ activation [99].	[99]
VCE-003.2	Enhances neuronal progenitor cell survival and to also provide a symptomatic relief in murine models of Huntington’s disease [101]. Showed protective effects in SOD1G93A transgenic mice, a model of amyotrophic lateral sclerosis [102].	[101,102]
HU-331	Anticancer properties [103]. Activation of the Keap1-Nrf2 pathway and BACH1 inhibitors [104].	[103,104]
Quinones **1**	Neuroprotective activity [105].	[105]
Asterriquinone B4	Neuroprotective activity in several cellular using oxidative stress models of Parkinson’s disease [106].	[106]
Asterriquinone 1H5 Indolylquinone 5E5	Promote neuronal differentiation of PC12 cells [107].	[107]
PQQ	Potent radical scavenger [113]. Reduces oxidative stress by increasing the expression of Nrf2 [115]. Neuroprotection of primary cultured hippocampal neurons against glutamate-induced cell damage by activation of the PI3K/Akt pathway [116]. Reduced indirectly the activity of GSK-3β [117]. Exerted effective protection in SH-SY5Y cells against 6-hydroxydopamine-induced neurotoxicity [113,114]. Reduce oxidative stress and improve mitochondrial functions and dopamine redistribution [118].	[113,114,115,116,117,118]
Geldanamycin	Binds to Hsp90 a target in neurodegeneration [119,120,121]. Protects against MPTP-induced dopaminergic neurotoxicity [123]. Rescues striatal dopamine levels [124]. Suppresses memory deficits in β-amyloid-injected rats [125]. Induces the degradation of misfolded tau protein [126]. Reduces brain injury in a mouse model of intracerebral hemorrhage and improves the neurological outcome of these mice [127].	[119,120,121,123,124,125,126,127]
Tanespimycin	Reduces polyglutamine-mediated motor neuron degeneration through proteasomal degradation of the androgen receptor in a mouse model [129]. Improves balance and coordination in a mouse model of the Machado-Joseph disease (spinocerebellar ataxia type 3) [130].	[129,130]
Rifampicin quinone	Inhibits fibrillation and disaggregates formed fibrils of β-amyloid [134]. Ameliorates the neurotoxic effects mediated by α-synuclein in microglial cells [136]. Protects PC12 cells against apoptosis induced by *N*-methyl-4-phenylpyridinium (MPP^+^). Suppresses the expression of α-synuclein multimers [139].	[134,136,139]
Thymoquinone	Protects against ischemic brain damage and reduces oxidative damage associated to epileptic seizures [142].	[142]
Juglone	Inhibits BACE and Aβ aggregation and induce Aβ fibril disaggregation [143]. Reverses the Thr-231 dephosphorylation of Tau induced by oxidative or heat stress in primary cortical cultures [145]. Neuroprotective potential by redox cycling [146] acting as a chelating agent [147]	[143,145,146,147]
Plumbagin	Reduces the extent of brain damage and neurological deficit associated to middle cerebral artery occlusion/reperfusion in mice [148]. Activates the Nrf2-ARE pathway, inhibits BACE-1 and attenuates astrocyte hyperactivation [149]. Inhibits the NF-kB pathway [150] and displays antioxidant and chelating activities [151].	[148,149,150,151]
Naphthoquinone **4**	Inhibits the aggregation of Aβ_40_ and the PHF6 tau fragment [152]. Inhibits AChE and MAO B [152].	[152]
Purpurin	Hampers the aggregation of PHF-6 and diassambles pre-formed PHF-6 fibrils, reducing Tau phosphorylation and accumulation [153]. Inhibits NF-κB activation [154]. Decreases oxidative stress and inflammation markers [155].	[153,154,155]
Emodin	Radical scavenger and chelating agent. Nrf2 inducer. Inhibits Aβ_42_ and Tau aggregation and toxicity. Inhibits AChE and BACE [152].	[152]
Rhein	Treatment of traumatic brain injury (TBI) by attenuating inflammation markers, pyroptosis and neurological dysfunction [158,160]. NADPH oxidase inhibition, avoiding the increase of ROS levels [159]. Modulation of inflammatory response by NF-kB-pathway and NLRP-3 inflammasome inhibition [161]. Moderate inhibition of AChE [183].	[158,159,160,161,183]
Compounds **5**	Neuroprotective activity against H_2_O_2_-induced oxidative stress. Reduces the levels of inflammatory cytokines and NO produced by macrophages exposed to LPS. Inhibits the production and aggregation of Aβ. Moderate AChE and BChE inhibition [164].	[164]
Quinones **6**	Inhibits the JAK-STAT pathway [165].	[165]
Memoquin	Scavenges ROS. Inhibitis β-amyloid aggregation, BACE-1 and AChE [174]. Prevents cognitive impairment [173].	[173,174]
Compound **7**	Inhibits BACE [175].	[175]
Compounds **8**	Inhibit ROS production and ROS-induced cytotoxicity. Induce Nrf2, thereby increasing the levels of the NQO1 enzyme [176]. Reduce Aβ_42_ self-aggregation. Inhibit AChE and BChE [177].	[176,177]
Compounds **9**	Inhibit β-amyloid aggregation Inhibit AChE [178].	[178]
Compounds **10**	Inhibit AChE. Inhibit spontaneous amyloid aggregation [179].	[179]
Compounds **11**	Inhibit AChE. Radical scavengers [180].	[180]
Compounds **12**	Selectively inhibit butyrylcholinesterase. Antioxidant and amyloid antiaggregating properties [181].	[181]
Compounds **13**	Inhibit both AChE and BChE. Inhibit β-amyloid aggregation [182].	[182]
Compounds **14**	Inhibit AChE [183].	[183]
Compounds **15**	Inhibit AChE and BChE. Inhibit BACE-1. Show dual Aβ42 and tau antiaggregating activity [185].	[185]
NQTrp	Reduces the accumulation of oligomeric Aβ species in an Alzheimer’s disease model of *Drosophila* flies [188]. Inhibits the aggregation of α-synuclein.	[188]
NQDA	Inhibits α-synuclein aggregation and disrupts preformed fibrils. Reduces aggregate-induced toxicity [189]	[189]

## Data Availability

Not applicable.
